# Measuring patient-perceived continuity of care for patients with long-term conditions in primary care

**DOI:** 10.1186/s12875-014-0191-8

**Published:** 2014-12-05

**Authors:** Kate M Hill, Maureen Twiddy, Jenny Hewison, Allan O House

**Affiliations:** Leeds Institute of Health Sciences, University of Leeds, Charles Thackrah Building, 101 Clarendon Road, Leeds, LS2 9LJ UK

**Keywords:** Continuity of care, Primary health care, Long-term conditions, Trust

## Abstract

**Background:**

Continuity of care is widely acknowledged as important for patients with multi-morbidity but simple, service-orientated indices cannot capture the full impact of continuity in complex care delivery systems. The patient’s perspective is important to assess outcomes fully and this is challenging because generic measures of patient-perceived continuity are lacking. We investigate the Chao Perception of Continuity (Chao PC) scale to determine its suitability as a measure of continuity of care for patients with a long-term condition (stroke), and co-morbidity, in a primary care setting.

**Methods:**

*Design and Setting:* A questionnaire study embedded in a prospective observational cohort study of outcomes for patients following acute stroke.

*Participants:* 168 community dwelling patients (58% male) mean age 68 years a minimum one year post-stroke. Functional status: Barthel Index mean =16.

*Intervention:* A 23-item questionnaire, the Chao Perception of Continuity (Chao PC) scale, sent by post to their place of residence or administered face to face as part of the final cohort study assessment.

**Results:**

310 patients were invited to participate; 168 (54%) completed a questionnaire.

All 23 questionnaire items were entered into a Principal Component Analysis. Emergent factors from the exploratory analysis were (1) inter-personal trust (relational continuity); (2) interpersonal knowledge and information (informational and relational continuity) and (3) the process of care (managerial continuity). The strongest of these was inter-personal trust.

**Conclusion:**

The context-specific items in the Chao PC scale are difficult for respondents to interpret in a United Kingdom Primary Care setting resulting in missing data and low response rates. The Chao-PC therefore cannot be recommended for wider application as a general measure of continuity of care without significant modification.

Our findings reflect the acknowledged dimensions of continuity and support the concept of continuity of care as a multi-dimensional construct. We demonstrate the overlapping boundaries across the dimensions in the factor structure derived. Trust and interpersonal knowledge are clearly identified as valuable components of any patient-perceived measure of continuity of care.

**Electronic supplementary material:**

The online version of this article (doi:10.1186/s12875-014-0191-8) contains supplementary material, which is available to authorized users.

## Background

Long term conditions present a major challenge for health care commissioners and providers in developed countries across the world. The number of patients with one or more chronic conditions is increasing, and their management can be complex. [1] Connecting care over time for patients with multi-morbidity can be difficult in health care systems that are largely organised around treatment for individual diseases and care can easily become fragmented. Notwithstanding the difficulties, it is important to maintain continuity of care for long-term conditions as it can lead to fewer complications and be more cost-effective [2]. In countries like the United Kingdom (UK) where Primary Care is well developed, the general practitioner is both the gateway to specialist services and the coordinator of care: referring patients at the outset; and monitoring them when they return to primary care for longer-term maintenance of care. Many of the pitfalls in continuity occur at the interfaces of care when management of care is transferred and information flow may be interrupted. Consequently much of the task of navigating the system and providing continuity of care for patients still remains with primary care practitioners.

Continuity of care is often described as the “cornerstone of care” and an “essential element” of general practice [3]. Early measurement of continuity of care was pursued largely from a service-orientated, clinician-centred perspective; simple indices were favoured and provider continuity (seeing the same doctor) was a persistent theme in assessment. Although that view of continuity is still important [4], there is a consensus that continuity of care is a multi-dimensional construct that needs to be considered both in the process of care and from the perspective of patients [5,6]. More recently, measures of continuity that encompass the multi-dimensionality of continuity of care have been developed and tested in the UK and the Netherlands [7,8]. The two examples quoted structured questionnaire items around two key, patient-centered dimensions of continuity: relational and informational continuity. We decided to test a questionnaire that measured continuity from the patient’s perspective using an earlier conceptual paradigm in order to explore the potential for other underlying constructs to emerge.

The Chao Perception of Continuity scale (Chao PC) [9] was inspired by the Banahans’ concept of continuity [10], which proposes continuity as an attitudinal contract residing in a two-way relationship between the patient and care provider based on the patient’s reliance on the doctor and the doctor’s responsibility and duty to the patient. Banahans’ attitudinal concept was not derived from empirical data or existing evidence but it did mark a key shift from simply counting provider-contacts to considering the patient-physician relationship in continuity. The Chao PC originated in the United States where the interface between primary and secondary care differs from the UK hence some items refer to hospital care. Given that care for long-term conditions is often shared between primary and secondary care we decided not modify the questionnaire for our study. The Chao-PC scale has not been widely used since its introduction but it was the first generic scale designed to measure continuity from the patients’ perspective and to “.....*provide information which is distinct from provider continuity formulas”*.

We are interested in assessing patient-reported continuity of care in long-term conditions that are often compounded by one or more co-morbidities. Associations between continuity of care, healthcare utilisation and patient satisfaction have been demonstrated but strong evidence of its effect on outcomes remains elusive and reviews report varying results [11,12]. Chao-PC was the only generic measure available that included relational items assessed from the patient’s perspective like trust in care providers and shared personal knowledge. Trust is recognised as an important factor in health care [13], and a connection between trust and interpersonal relationships has been demonstrated but we do not fully understand how patients value their personal connections with care providers or what trade-offs they will make for access to care [14]. It is therefore important to develop reliable methods of measuring continuity that take account of the way complex care is delivered. This paper re-examines the underlying constructs of the Chao PC scale and tests its discriminatory properties to determine its suitability as a measure of patient-perceived continuity.

## Methods

### Participants

We recruited 310 stroke survivors living in the community approximately one year post-acute stroke from a cohort of patients participating in the Stroke Outcomes Study (SOS2). SOS2 was a prospective longitudinal cohort study that examined the impact of early depressive symptoms on outcomes for patients in the year after stroke. A range of physical and psychosocial outcomes assessments were collected at the time of recruitment to the study and at four follow-up points. The SOS2 study protocol has been fully described elsewhere [15].

Ethical approval for the study reported in this paper was granted by the NRES Leeds (East) Research Ethics Committee; Reference Number: 02/09/236.

### Measures

The Chao PC scale consists of a self-administered questionnaire containing 23 items organised in two sections: Section 1 items assess the care process, which Chao described as objective items; and Section 2 the relational (or remaining) dimensions of continuity. Items are scored on a standard Likert type 5-point scale. Section 1 items are rated ’definitely true; mostly true; uncertain; mostly false or definitely false’ (scored 1 to 5). Section 2 items are rated from ‘agree strongly’ to ‘disagree strongly’ on the same scoring scale. For each subscale, a higher score relates to higher perceived continuity.

The Chao PC scale (see Additional file 1: Appendix A) was originally tested in a small study in the USA (n = 147) [9]. Initial results were promising, with high internal consistency and reliability reported. The reported principal component analysis revealed two constructs; one related to the process of healthcare delivery and the other related to the interpersonal relationship between the patient and physician. The two constructs encompassed items that reflected the layout of the questionnaire, which is organised in two sections. Dual loading was allowed on factors. External validity was assessed against the Continuity of Care (COC) Index [16] and the Usual Provider Continuity (UPC) Index [17] but only a modest association was found with these two measures. This is not surprising given that the COC and the UPC measure only the proportion of contacts with the same care provider (provider continuity) and not the wider dimensions of continuity that the items in the Chao-PC encompass.

Frequency and duration of healthcare contact is influenced by severity of physical illness and by associated emotional disorder. In order to assess the influence of these moderators of response in our study, our participants completed a number of additional measures: the General Health Questionnaire (GHQ-28) [18] a measure of psychological wellbeing (mood), the Functional Independence Measure [19] and the Barthel Index [20], which are self-report measures of functional capacity and level of dependency. The GHQ was scored using the modified dichotomous scoring method (c-GHQ: 0-1-1-1) [21]; the recommended cut-off point of >11 was taken as indicative of psychological distress [22].

### Study procedures

All measures were administered to 110 participants in the cohort study who agreed to complete it as part of their final SOS2 follow-up visit, conducted one year after their index stroke event. Face to face delivery enabled the applicability and understanding of questionnaire items to be checked in a UK population. Thereafter the Chao PC scale was sent by post to 200 patients who had already completed their final SOS2 study assessment. Consent to further contact had already been obtained from patients at the end of the cohort study thus return of the completed questionnaire was deemed to indicate consent to participate. A stamped addressed envelope was included in the mailing for return of the completed questionnaire. Responses were anonymised but could be linked to demographic and outcome data from the cohort study through a unique identification code.

The first interviewed sample showed that the ‘*uncertain’* label for the mid-range value was frequently used by responders to indicate uncertainty about the meaning of the question rather than uncertainty about their response. Consequently an additional missing value option for ‘*not applicable’* was included in the postal versions to reduce the chances of a respondent assigning a mid-range score to a ‘*not applicable*’ rating. Items scored ‘*not applicable’* were coded as *‘missing’.*

### Data analysis

SPSS Version 18 software was used to analyse the data [23]. Reverse scored items (items: 1b, 1d, 1f, 1 h, 2a, 2d-e, 2 h, 2j-o) were rescaled, a higher score thus always indicated greater patient-perceived continuity. No weighting was applied to items. Descriptive statistics were calculated for all items (means and standard deviation). Five questionnaires had insufficient data and were excluded from the analysis. The Kaiser-Meyer-Olkin statistic was calculated (KMO =0.74) indicating that Principal Component Analysis (PCA) was appropriate.

We chose an exploratory rather than confirmatory approach to the analysis because our sample differed significantly from the younger primary care sample studied by Chao [9]. As a first step we sought to determine whether the original Chao PC two-factor structure could be replicated in our sample using exploratory Principal Components Analysis (PCA). We then modelled our data a second time using PCA and derived a factor structure emergent from our sample without reference to the Chao model. In each case Varimax rotation was applied and the Kaiser criterion (eigenvalues >1) was used to determine the factor structure. To reflect the sample size, only factor loadings greater than 0.51 were considered significant [24].

## Results

Data were collected from 108 of the 110 patients approached at interview (98%). Only two participants declined to complete the questionnaire in this supported setting, although a number rated items *‘Not Applicable’*. Data retrieved from the postal questionnaires were less comprehensive: Sixty questionnaires were returned fully completed out of the 200 mailed to participants (Response rate: 30%). This gave a total of 168 (98 (58%) male; 69 (42%) female and 1 unknown gender) questionnaires for analysis, representing 54% of the total eligible population (N =310). Demographic data could not be linked for one respondent in the postal sample due to a clerical error.

### Participant characteristics

Table 1 shows the characteristics of the participants (n =168); all of whom were living at home, under the care of their General Practitioner; only two were being followed-up by a stroke specialist. One hundred and two patients in the sample were aged between 65 and 84. In this older group over half (58%) had two or more physical or mental health disorders [mean 2.2 (SD 1.9)]; the younger participants (<65 yrs old; n =59) had fewer [29%; mean 1.7(SD 1.34)]. Our sample characteristics are comparable with the larger sample of general primary care patients of similar age range described by Barnett et al. [1].

**Table 1:** **Characteristics of our study participants**

**Patient characteristics**	**N = 168**
***Values are n (%) unless otherwise stated***	
**Mean age, years (Mean SD)**	67.65 (12.54)
Male	N = 98 (58.3)
**Ethnicity**	
White British	165 (98.2)
**Marital status**	
Married/co-habiting	103 (61.3))
Single	14 (8.3)
Widowed	34 (20.2)
Divorced/separated	13 (9.5)
Not reported	1 (0.6)
**Educational level**	
< 16 yrs.	126 (79)
< 18 yrs.	24 (14.3)
>18 yrs	10(6)
**Multi-morbidity**	107 (67.3%)
Number of morbidities [Mean (SD)]	2.22 (1.96)
GHQ 28 total >11	37 (22)
GHQ28 Total [Mean (SD)]	7.17 (6.02)
Barthel Index [Mean (SD)]	16.13 (4.82)
Functional independence measure (FIM) [Mean (SD)]	116.1 (15.87)

Twenty-two percent of the total sample scored above the cut-off (>11) on the GHQ-28 for ‘caseness’ which was slightly lower than the average proportion estimated in other observational studies in stroke [25]. Postal and interview sample participants were compared for characteristics that could be a source of bias. No significant differences were found.

### Descriptive statistics for Chao PC

Descriptive statistics for each variable (Table 2) are presented as number and percent of respondents or mean and standard deviation (SD). The overall mean score of the Chao-PC scale in our sample was 3.7 (SD 0.6; range 2.2 to 5.0), which is comparable with that found by Chao in the original study (3.6; SD 0.6; range 2.2 to 4.9). Data distributions in the item and total score were skewed, with a ceiling effect evident in both datasets.

**Table 2:** **Descriptive statistics for the Chao PC scale items**

**Chao item no.**	**Variable descriptor**	**N**	**Mean**	**Std. deviation**
1A	Different doctors	133	3.43	1.755
1B	Past medical problems	151	2.75	1.488
1C	Location	155	3.18	1.749
1D	Medication	161	4.80	.603
1E	Same doctor	160	3.59	1.510
1 F	Prior knowledge	153	3.43	1.255
1G	Unknown problems	159	4.55	.979
1H	Care for all problems	158	3.91	1.274
2A	On-going relationship	159	3.77	1.317
2B	Unrelated medical problems	152	3.77	1.242
2C	Personal problems	154	3.68	1.441
2D	Knowledge of family members	148	3.20	1.419
2E	Ease of communication	160	4.19	1.084
2 F	Knowledge of family problems	132	3.32	1.355
2G	Poor explanations	157	3.83	1.325
2H	Emergency care preference	156	3.36	1.532
2I	Waiting for own doctor	152	2.73	1.409
2 J	Referrals	157	4.18	.951
2 K	Provides pre-admission care	151	2.91	1.282
2 L	Provides ER care	155	3.50	1.261
2 M	Trust recommendations	159	4.35	.763
2 N	Recognition	161	3.50	1.383
2O	Trust personal	158	4.39	.895
	Total score	168	3.72	0.57

### Exploratory PCA: validating the 2 factor model

To validate the Chao PC in a UK population all 23 items were entered into a PCA and an *a priori* 2 Factor-solution was applied to the data. The proportion of variance explained was modest (32.52%). There was poor correspondence between the models derived from the analysis conducted by Chao [9] and the current analysis (Table 3); only 4 items were found in common. These are highlighted in Table 3.

**Table 3:** **Results of the principal component analysis (2 factor solution)**

**Chao item no.**	**Variable descriptor**	**Component**	
		**1**	**2**
1A	Different doctors	.486	
1B	Past medical problems		.600
1C	Location		-.402
1D	Medication		.456
1E	Same doctor	.431	
1 F	**Prior knowledge**		**.428**
1G	Unknown problems	.483	
1H	Care for all problems	.480	.474
2A	On-going relationship	.454	.579
2B	**Unrelated medical problems**	**.558**	
2C	Personal problems		
2D	Knowledge of family members	.418	.430
2E	Ease of communication	.525	
2 F	Knowledge of family problems	.579	
2G	Poor explanations	.647	
2H	Emergency care preference		.461
2I	Waiting for own doctor		
2 J	**Referrals**		**.514**
2 K	Provides pre-admission care		.483
2 L	**Provides ER care**		**.577**
2 M	Trust recommendations	.410	.428
2 N	Recognition	.424	.519
2O	Trust personal	.558	.504

Overlapping items are highlighted in bold type.

Extraction Method: Principal Component Analysis. Rotation Method: Varimax with Kaiser Normalization.

Rotation converged in 3 iterations.

### Exploratory analysis: 7 factor solution

A second PCA was conducted to examine the factor structure of the dataset. Due to the exploratory nature of the analysis, no limit was placed on the number of factors to be extracted. Table 4 shows the factor loadings of this second analysis. It resulted in seven factors explaining 61.5% of the total variance.

**Table 4:** **Results of the principal component analyses**

	**Factor 1**	**Factor 2**	**Factor 3**	**Factor 4**	**Factor 5**	**Factor 6**	**Factor 7**
**Eigenvalue**	5.52	1.96	1.76	1.41	1.23	1.19	1.01
Have an on-going doctor-patient relationship (2A)*	0.52						
Get appropriate referrals (2J)	0.73						
Trust a recommended specialist (2M)	0.728						
Trust my Doctor (2O)	0.714						
Doctor knows about my family members (2D)		0.654					
Doctor knows about family problems (2 F)		0.827					
Doctor explains things to me (2G)		0.597					
Care for all problems (1H)			0.589				
Would provide care if going to hospital (2K)			0.788				
Would provide care in an emergency (2L)			0.759				
Past medical problems (1B)*				0.64			
Medication (1D)				0.55			
Care improves with provided continuity (1F)*				0.64			
Waiting for own doctor (2I)*					0.86		
Doctor would know me on the street (2N)*					0.52		
Unrelated medical problems (2B)*						0.72	
Personal problems (2C)*						0.83	
We go to different doctors (1A)*							0.51
Medical problems doctors not know about (1G)*							0.69

*Denotes items removed following Reliability Analysis.

Values below 0.5 have been suppressed and missing values were excluded listwise.

Extraction Method: Principal Component Analysis. Rotation Method: Varimax with Kaiser Normalization.

Rotation converged in 12 iterations.

### Reliability

Factor analysis aims to reduce the observed data to uncover underlying concepts but defining a true model can be difficult. To determine the reliability of our model we examined the inter-item correlations and Cronbach α for the Chao PC subscales. Mean inter-item correlation of the subscales varied between *r =*0.3 and *r* =0.6. Internal consistency (Cronbach α) ranged from 0.7 to 0.76. We removed items with item-total correlation values less than 0.2 and the reduced data set was re-modelled using PCA.

A conventional value of factor weight >0.51 for a sample greater than 100 was taken as significant, and values below this were suppressed [24]. This resulted in a three factor solution that explained 68% of the variance and that mapped to the Banahans’ concept of continuity and to the process of care. Table 5 shows that variables related to trust and inter-personal relationships load substantially onto Factor 1. Factor 2 includes items relating to inter-personal knowledge and information, whilst Factor 3 includes items relating to medical care processes.

**Table 5:** **Final factor solution**

	**Component**		
	**Factor 1:**	**Factor 2:**	**Factor 3:**
	**Interpersonal trust**	**Interpersonal knowledge and information**	**Provides consistent care**
	**(Patient to Doctor)**	**(Doctor to Patient)**	
	**α = 0.76**	**α =0.7**	**α =0.72**
Get appropriate referrals (2 J)	**.813**	.066	.126
Trust a recommended specialist (2 M)	**.805**	.127	.136
Trust my doctor (2O)	**.691**	.395	.105
Doctor knows about my family members (2D)	.133	**.797**	.108
Doctor knows about family problems (2 F)	.086	**.884**	-.014
Doctor explains things to me (2G)	.241	**.630**	.193
Care for all problems (1H)	.494	.258	**.537**
Would provide care if going to hospital (2 K)	.384	.095	**.757**
Would provide care in an emergency (2 L)	-.035	.063	**.893**

Higher factor loadings are indicated in bold type.

Extraction Method: Principal Component Analysis. Rotation Method: Varimax with Kaiser Normalization.

Rotation converged in 5 iterations.

We considered the moderating effect that the psychological status of our participants might have on the data and examined this using the GHQ-28. Distressed patients reported lower levels of ‘Interpersonal Trust’, higher levels of ‘Interpersonal Knowledge’, and a belief that their doctor provided consistent care but these differences did not reach significance (p > 0.05). We also hypothesised that disability could be a driver of care patterns but no significant differences were found based on the measures of functional status (all p > 0.05).

## Discussion

Patients with multi-morbidity or long-term conditions may be under the care of several specialists in secondary care at various points in time; each of whom sends information separately about their management to the GP by means of discharge notes or clinical letters. Within the hospital system, information transfer between specialties relies mainly on patient records. Providing high quality, holistic, longitudinal care in a complex healthcare system can be challenging particularly for patients with both physical and mental health problems [26]. Multidisciplinary teams go some way to addressing this but records can be lost or inaccessible particularly where care crosses different sectors of the health system. The management and coordination of care for people with long-term conditions and multi-morbidity therefore increasingly resides in primary care, and continuity of care is essential if seamless care is to be delivered and outcomes for patients are to be improved.

Moreover, the burden on patients coping with multiple health problems needs to be acknowledged and it is important to understand how patients experience continuity in their care in order to maintain and monitor standards, and improve care quality. Measurement of continuity of care from the patient’s perspective has been hindered in the past by a lack of conceptual clarity, and there is still considerable debate about the distinction between continuity and coordination [27]. In this study we tested the factorial validity and structure of an existing measure of patient-perceived continuity, the Chao PC, in a UK population to determine its suitability as a general measure of continuity in primary care. The major construct to emerge from our analyses related to ‘*Interpersonal Trust’*; a core component of the doctor-patient relationship. The second factor related to ‘*Interpersonal Knowledge’* and incorporated questions related to how much the doctor knows about the patient and doctor-patient communication, and the third encompassed structural elements of ‘*Medical Care’,* including issues relating to the provision of care by the GP. We found that Factors 1 and 3 from our study closely mirrored Chao’s Factor 1: ‘*Structural Elements*’. The items loading to our Factor 2 (*Interpersonal Knowledge*) reflected Chao’s Factor 2 referred to as *Interpersonal Elements*. However, Chao identified more items loading to interpersonal elements of care than we found in our study.

Trust and interpersonal knowledge emerged as major factors in both analyses. Trust is an important attribute of medical care and evidence shows that it can influence patients’ engagement with care processes and their satisfaction with care [13,28]. It is an important basis for establishing confidence in health care professionals and is one way in which patients may experience continuity of care directly [29]. We can therefore offer an explanation of the link between trust and interpersonal knowledge evident in our findings given that trust develops over time, growing as relationships with care providers become more established and in turn making it more likely that such relationships will be sustained and contribute to better continuity of care.

The external validity of the Chao PC was originally tested against the Continuity of Care and Usual Provider Continuity indices but only a modest association was found [9]. In our study we found that the Chao was not sensitive to mood or functional status, factors that might be expected to drive care patterns and consequently to influence the personal experience of continuity of care.

### Limitations of the study

Participants in this study were drawn from a cohort of stroke patients; our sample is therefore older than a typical primary care sample but the comparison with a nationally representative sample of patients of similar age range suggests that we have captured one of primary care’s major user groups: older people with a chronic condition compounded by co-morbidity [1]. It is also arguable that our sample is more representative than the sample in the original Chao study which was young, affluent and educated.

We found two questions *“we go to different doctors”* (1a) and *“knowledge of family members”* (2d) were not relevant to responders in this study. Even within families, individuals did not necessarily attend the same GP surgery. This is not a specific feature of our stroke sample and could occur in any primary care sample from a UK population. Items that clearly reflected the US context in which the measure was developed (for example 2 k and 2 l) also caused difficulty with interpretation. Full details of questionnaire items are reproduced in Additional file 1: Appendix A.

To pre-empt the difficulties in interpretation we had identified in the face to face interviews, we added a new category ‘*not applicable’* to the postal questionnaires and controlled for its effect by factor analysing the two samples separately. No differences in the overall factor structure were found. We also removed data from the main analysis for five respondents who returned questionnaires with more than five missing items. Face to face data collection was clearly the most effective method of reducing missing data enabling queries to be resolved during completion of the questionnaire. The drawback is research-time costs compared to postal distribution of questionnaires.

## Conclusion

This study has demonstrated that Chao PC is not easily transferred to a UK primary care setting. The main drawback is the style of the questions, which cause confusion between primary and secondary care processes. The original questionnaire cannot therefore be recommended for wider use outwith its original context unless it was subject to significant modification. Nevertheless, questions about trust and interpersonal knowledge did emerge as relevant and could be considered valuable components of any patient-perceived measure of continuity of care.

The strong representation of trust in the two separate analyses suggests that trust in health care providers is a robust concept. A common theme in many definitions of trust in a medical context is vulnerability, characterised by an expectation that healthcare professionals will do the right thing medically. There is an asymmetry in personal relationships inherent in this account which marks a difference from the role of trust usually posited in social capital, where trust is reinforced by a social contract based upon ties of mutual expectation and obligation. This has implications for continuity of care as we can hypothesise that building trust in medical care could improve continuity of care. The means to do that will differ according to whether we are attempting to build trust at an individual (practitioner) or organisational (health service) level, but will surely entail a sustained effort to demonstrate willingness and competence to act in the patient’s best interest when they are vulnerable, rather than simply to provide a contracted-for service. Assessing the impact of interventions designed to increase trust is complicated by the difficulty of measuring patient-perceived continuity and the complex array of factors that influence patients’ experience of care.

## Additional file

Additional file 1:
**Appendix A: The Chao Continuity Questionnaire.**

## References

[1] Barnett K, Mercer SW, Norbury M, Watt G, Wyke S, Guthrie B (2012). Epidemiology of multimorbidity and implications for health care, research, and medical education: a cross-sectional study. Lancet.

[2] Hussey PS, Schneider EC, Rudin RS, Fox D, Lai J, Pollack C (2014). Continuity and the costs of care for chronic disease. JAMA Intern Med.

[3] Freeman GK, Olesen F, Hjortdahl P (2003). Continuity of care: an essential element of modern General Practice?. Fam Pract.

[4] Nyweide DJ, Anthony DL, Bynum JP, Strawderman RL, Weeks WB, Casalino LP, Fisher ES (2013). Continuity of Care and the Risk of Preventable Hospitalization in Older Adults. JAMA Intern Med.

[5] Freeman G, Shepperd S, Robinson I, Ehrich K, Richards S, Pittman P (2000). Continuity of care: report of a scoping exercise for the SDO programme of NHS R&D.

[6] Reid RJ, McKendry R, Haggerty J (2002). Defusing the Confusion: Concepts and Measures of Continuity of Health Care: Final Report.

[7] Uijen AA, Schellevis FG, van den Bosch WJHM, Mokkink HGA, van Weel C, Schers HJ (2011). Nijmegen Continuity Questionnaire: development and testing of a questionnaire that measures continuity of care. J Clin Epidemiol.

[8] Gulliford M, Cowie L, Morgan M (2011). Relational and management continuity survey in patients with multiple long-term conditions. J Health Serv Res Policy.

[9] Chao J (1988). Continuity of care: incorporating patient perceptions. Fam Med.

[10] Banahan BFJ, Banahan BFI (1981). Continuity as an Attitudinal Contract. J Fam Pract.

[11] Van Walraven C, Oake N, Jennings A, Forster AJ (2010). The association between continuity of care and outcomes: a systematic and critical review. J Eval Clin Pract.

[12] Adler R, Vasiliadis A, Bickell N (2010). The relationship between continuity and patient satisfaction: a systematic review. Family Practice, Volume 27.

[13] Trachtenberg FDE, Hall MA (2005). How patients’ trust relates to their involvement in medical care. J Fam Pract.

[14] von Bültzingslöwen I, Eliasson G, Sarvimäki A, Mattsson B, Hjortdahl P (2006). Patients’ views on interpersonal continuity in primary care: a sense of security based on four core foundations. Fam Pract.

[15] Hill K, West R, Hewison J, House A (2009). The Stroke Outcomes Study 2 (SOS2): a prospective, analytic cohort study of depressive symptoms after stroke. BMC Cardiovasc Disord.

[16] Bice TW, Boxerman SB (1977). A Quantitative Measure of Continuity of Care. Med Care.

[17] Breslau N (1982). Continuity Reexamined: Differential Impact on Satisfaction with Medical care for Disabled and Normal Children. Med Care.

[18] Goldberg DP, Hillier VF (1979). A scaled version of the General Health Questionnaire. Psychol Med.

[19] Hamilton BB, Laughlin JA, Fielder RC, Granger CV (1994). Inter-rater reliability of the 7-level Function Independance Measure. Scan J Rehabil Med.

[20] Mahoney F, Bartel D (1965). Functional Evaluation: the Bartel Index. Md State Med J.

[21] Huppert F, Gore M, Elliott B (1988). The value of an improved scoring system (C-GHQ) for the General Health Questionnaire in a representative community sample. Psychol Med.

[22] Goldberg D, Gater R, Sartorius N, Ustun T, Piccinelli M, Gureje O, Rutter C (1997). The validity of two versions of the GHQ in the WHO study of mental illness in general health care. Psychol Med.

[23] SPSS I (2009). PASW Statistics for Windows, Version 18.0.

[24] Stevens J (1992). Applied multivariate statistics for the social sciences.

[25] Hackett ML, Yapa C, Parag V, Anderson CS (2005). Frequency of depression after stroke: a systematic review of observational studies. Stroke.

[26] Mercer SW, Gunn J, Bower P, Wyke S, Guthrie B (2012). Managing patients with mental and physical multimorbidity. BMJ.

[27] Chen LM, Ayanian JZ (2014). Care continuity and care coordination: What counts?. JAMA Intern Med.

[28] Safran DG, Taira DA, Rogers WH, Kosinski M, Ware JE, Tarlov AR (2002). Linking primary care performance to outcomes of care. J Fam Pract.

[29] Haggerty JL, Roberge D, Freeman GK, Beaulieu C (2013). Experienced continuity of care when patients see multiple clinicians: a qualitative metasummary. Ann Fam Med.

