# Supplementary material for: Measuring patient-perceived continuity of care for patients with long-term conditions in primary care
Source: BMC Fam Pract. 2014 Dec 5;15:191. doi: 10.1186/s12875-014-0191-8 (PMC4264317; doi:10.1186/s12875-014-0191-8)
Supplement: Additional file 1: — Appendix A: The Chao Continuity Questionnaire. [file 12875_2014_191_MOESM1_ESM.pdf]

Appendix A:

THE CHAO CONTINUITY QUESTIONNAIRE

© Dr. Jason Chao M.D., M.S. Associate Professor of Family Medicine, Case Western Reserve University, Cleveland, Ohio.

1. PLEASE USE A SCALE OF 1 TO 5 TO INDICATE HOW TRUE OR FALSE YOU FEEL EACH OF THE FOLLOWING STATEMENTS IS:

DEFINITELY TRUE	MOSTLY TRUE	UNCERTAIN	MOSTLY FALSE	DEFINITELY FALSE
1	2	3	4	5

Please write your score in the box next to the question	
A. If more than one family member needs medical care, we go to different doctors.	
B. My doctor often mentions or refers to my past medical problems and treatments.	
C. I receive my medical care at more than one location.	
D. The doctor has a list of all the medicines which I am taking now.	
E. I rarely see the same doctor when I go for medical care.	
F. My medical care improves when the doctor has seen me before.	
G. I have medical problems that the doctor doesn't know about.	
H. My doctor provides care for any type of problem which I may have.	

2. PLEASE INDICATE YOUR OPINION OF THE FOLLOWING STATEMENTS, USING THE SCALE 1 TO 5:

AGREE STRONGLY	AGREE	UNCERTAIN	DISAGREE	STRONGLY DISAGREE
1	2	3	4	5

Please write your score in the box next to the question	
A. I feel that I have an on-going relationship with a doctor.	
B. It is difficult to bring up unrelated medical problems with the doctor.	
C. I am uncomfortable in discussing a personal problem with the doctor.	
D. The doctor knows a lot about the rest of my family.	
E. I feel comfortable asking questions of the doctor.	
F. The doctor doesn't know about my family problems.	
G. The doctor does not explain things to me.	
H. In an emergency, I want my regular doctor to see me.	
I. I would rather see another doctor right away, instead of waiting a day or two to see my regular doctor.	
J. My doctor provides appropriate referrals to other specialists.	
K. My doctor would take care of me if I had to go to the hospital.	
L. My doctor would take care of me if I require emergency care.	
M. I trust a specialist recommended by my doctor.	
N. My doctor would know me by name if we met on the street.	
O. I trust my doctor.	
